# Draft Genome Sequence of 2-Methylpyridine-, 2-Ethylpyridine-, and 2-Hydroxypyridine-Degrading *Arthrobacter* sp. Strain ATCC 49987

**DOI:** 10.1128/MRA.00748-20

**Published:** 2020-08-20

**Authors:** Nidhi Gupta, Kelly A. Skinner, Zarath M. Summers, Janaka N. Edirisinghe, José P. Faria, Christopher W. Marshall, Anukriti Sharma, Neil R. Gottel, Jack A. Gilbert, Christopher S. Henry, Edward J. O’Loughlin

**Affiliations:** a Argonne National Laboratory, Lemont, Illinois, USA; b University of Chicago, Chicago, Illinois, USA; c ExxonMobil Research and Engineering Company, Annandale, New Jersey, USA; University of Southern California

## Abstract

Here, we report the draft genome sequence of *Arthrobacter* sp. strain ATCC 49987, consisting of three contigs with a total length of 4.4 Mbp. Based on the genome sequence, we suggest reclassification of *Arthrobacter* sp. strain ATCC 49987 as *Pseudarthrobacter* sp. strain ATCC 49987.

## ANNOUNCEMENT

*Arthrobacter* spp. are ubiquitous members of soil microbial communities that play important roles in biogeochemical cycling and bioremediation, and many have been studied for their ability to grow under different environmental stresses to degrade persistent environmental pollutants (1, 2). O’Loughlin et al. isolated a 2-methylpyridine-degrading bacterium (*Arthrobacter* sp. strain ATCC 49987) from subsurface sediment from an aquifer located at an industrial site that had been contaminated with pyridine and pyridine derivatives (3). In addition to 2-methylpyridine, ATCC 49987 utilizes 2-ethylpyridine and 2-hydroxypyridine as primary nitrogen, carbon, and energy sources (3). The genome sequence reported here will help us understand the genetic basis of the biotransformation of these compounds.

ATCC 49987 was obtained from the American Type Culture Collection (ATCC) and grown in tryptic soy broth for 16 h at 30°C. DNA was extracted using the Qiagen DNeasy PowerSoil genomic DNA extraction kit (product number 12888-50) as per the manufacturer’s instructions. DNA libraries were prepared using the Oxford Nanopore LSK109 kit for GridION X5 sequencing and the Nextera XT paired-end kit (Illumina) for Illumina MiSeq sequencing. For the GridION reads, base calling was carried out using Guppy v3.2.6, followed by adaptor removal and demultiplexing using Porechop v0.2.3. Paired-end reads (10,004,660 paired-end reads with an average length of 151 bp) along with single-end reads (331,392 single-end reads with an average length of 11,799 bp) were uploaded into the U.S. Department of Energy KBase system (https://kbase.us/n/64079/39) for further analysis (4). All bioinformatic tools were used with default parameters unless stated otherwise. FastQC v0.11.5 was used to assess read quality (5), and sequences were assembled using the MaSuRCA assembler v3.2.9 (6). The draft assembly had three contigs, with the largest consisting of 4,169,727 bp and the other two containing 65,535 bp and 167,082 bp. The total size of the resulting draft genome was 4.4 Mbp, with a GC content of 66.62% and genome coverage of 1,231×. The quality of assembly was checked using CheckM v1.0.18 (7), and the assembly was determined to be 99.7% complete, with 1.17% contamination. Annotation was completed using Prokka v1.12 (8), revealing 4,153 total genes, 66 noncoding RNAs, and 77 noncoding repeats (https://narrative.kbase.us/#dataview/64079/42/1). ATCC 49987 is reported to utilize 2-, 3-, and 4-hydroxybenzoate, gentisic acid, protocatechuic acid, and catechol in addition to pyridine derivatives (2-methylpyridine, 2-ethylpyridine, and 2-hydroxypyridine) (3), suggesting that it has broad potential for the biotransformation of a range of monoaromatic compounds. In-depth analysis of the genome of ATCC 49987 will further our understanding of the underlying mechanisms, thus guiding genetic modification of pathways to enhance its biotransformation capabilities.

**FIG 1:**
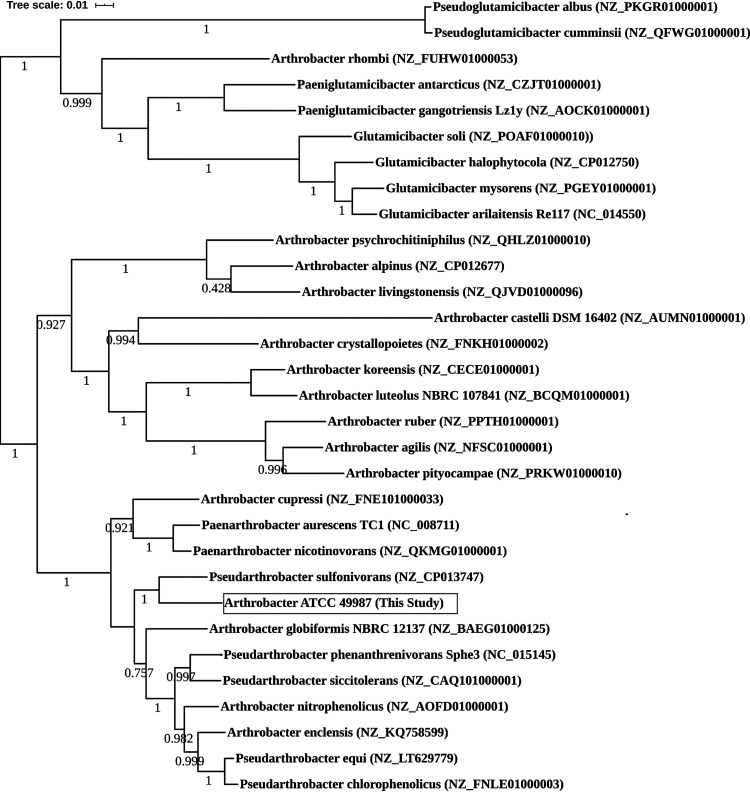
Phylogeny of ATCC 49987 in comparison with different species of *Arthrobacter*, *Paenarthrobacter*, *Glutamicibacter*, *Pseudarthrobacter*, *Pseudoglutamicibacter*, and *Paeniglutamicibacter* (RefSeq accession numbers in parentheses) selected from public KBase genomes. The whole genomes were aligned and the approximately maximum likelihood tree of ATCC 49987 was constructed using the default settings of FastTree v2.1.10 (10). Local support values for the tree nodes were computed using the Shimodaira-Hasegawa test in FastTree v2.1.10 and are represented on the tree.

At the time of isolation, ATCC 49987 was identified as a member of the genus *Arthrobacter* based on its physiological and morphological characteristics (3). However, certain species of the genus *Arthrobacter* have been reclassified into five novel genera, namely, *Pseudoglutamicibacter*, *Pseudarthrobacter*, *Glutamicibacter*, *Paeniglutamicibacter*, and *Paenarthrobacter* (9). To assess the relatedness of the ATCC 49987 genome, a tree was generated in KBase using the application “insert set of genomes into species tree.” The application first ran RPS-BLAST v0.3.3 with 49 universal conserved marker genes. The marker genes were selected based on the Clusters of Orthologous Groups (COGs) from NCBI (https://github.com/kbaseapps/SpeciesTreeBuilder/tree/master/data/cogs) against the ATCC 49987 genome. The RPS-BLAST alignments of marker genes generated using the draft genome and 30 closely related RefSeq genomes were concatenated, and then a tree was constructed using FastTree v2.1.10 with default settings (10) (Fig. 1). *Arthrobacter* sp. strain ATCC 49987 is most closely related to Pseudarthrobacter sulfonivorans; therefore, we suggest reclassification of *Arthrobacter* sp. strain ATCC 49987 as *Pseudarthrobacter* sp. strain ATCC 49987.

### Data availability.

The whole-genome sequences have been deposited in GenBank under accession numbers JAABNS010000001 through JAABNS010000003, and the SRA accession numbers are SRR10884004 and SRR10884005. The assembly with annotation can be accessed at NZ_JAABNS010000001, NZ_JAABNS010000002, and NZ_JAABNS010000003.
